# Re-assessment of the subcellular localization of Bazooka/Par-3 in *Drosophila*: no evidence for localization to the nucleus and the neuromuscular junction

**DOI:** 10.1242/bio.060544

**Published:** 2024-06-27

**Authors:** Soya Kim, Jaffer Shahab, Elisabeth Vogelsang, Andreas Wodarz

**Affiliations:** ^1^ Molecular Cell Biology, Center for Anatomy, University of Cologne and University Hospital Cologne, Weyertal 115c, 50931 Köln, Germany; ^2^ Stem Cell Biology, Institute for Anatomy and Cell Biology, Georg-August-University Göttingen, Justus-von-Liebig-Weg 11, 37077 Göttingen, Germany; ^3^ Center for Molecular Medicine Cologne, University of Cologne and University Hospital Cologne, Robert-Koch-Str. 21, 50931 Cologne, Germany; ^4^ Cluster of Excellence - Cellular stress response in aging-associated diseases (CECAD), University of Cologne and University Hospital Cologne, Joseph-Stelzmann-Str. 26, 50931 Cologne, Germany

**Keywords:** Bazooka/Par-3, Nuclear localization, Neuromuscular junction, Subcellular localization

## Abstract

Bazooka/Par-3 (Baz) is an evolutionarily conserved scaffold protein that functions as a master regulator for the establishment and maintenance of cell polarity in many different cell types. In the vast majority of published research papers Baz has been reported to localize at the cell cortex and at intercellular junctions. However, there have also been several reports showing localization and function of Baz at additional subcellular sites, in particular the nuclear envelope and the neuromuscular junction. In this study we have re-assessed the localization of Baz to these subcellular sites in a systematic manner. We used antibodies raised in different host animals against different epitopes of Baz for confocal imaging of *Drosophila* tissues. We tested the specificity of these antisera by mosaic analysis with null mutant *baz* alleles and tissue-specific RNAi against *baz*. In addition, we used a GFP-tagged gene trap line for Baz and a bacterial artificial chromosome (BAC) expressing GFP-tagged Baz under control of its endogenous promoter in a *baz* mutant background to compare the subcellular localization of the GFP-Baz fusion proteins to the staining with anti-Baz antisera. Together, these experiments did not provide evidence for specific localization of Baz to the nucleus or the neuromuscular junction.

## Teaser

**Summary:** The authors found that Bazooka/Par-3 does not localize to the nucleus or the neuromuscular junction, questioning earlier findings reporting a function of Baz at these subcellular sites.

## INTRODUCTION

Baz/Par-3 is a multi-domain scaffold protein with well-characterized roles in the establishment and maintenance of cell polarity. Baz is a core member of the polarity regulating Par complex, which it forms together with its evolutionarily conserved binding partners aPKC and Par-6 (Goldstein and Macara, 2007; Johnson and Wodarz, 2003; St Johnston and Ahringer, 2010; Tepass, 2012). Baz/Par-3 was first identified in *C. elegans* (Etemad-Moghadam et al., 1995) with the subsequent identification of homologues in *Drosophila* (Kuchinke et al., 1998), and mammals (Izumi et al., 1998). In *C. elegans*, Baz/Par-3 functions as a key regulator of spindle orientation and polarity in the early embryo (Etemad-Moghadam et al., 1995) and the assembly of adherens junctions in the intestine (Achilleos et al., 2010). In *Drosophila*, Baz has been shown to function as a key regulator of apico-basal polarity in epithelial cells (Harris, 2012; Tepass, 2012). Baz functions at the top of a genetic hierarchy in the establishment of adherens junctions during cellularization (Harris and Peifer, 2004, 2005) and in the maintenance of apico-basal polarity during embryonic epithelial development (Bilder et al., 2003; Müller and Wieschaus, 1996; Tanentzapf and Tepass, 2003). Baz is also required for oocyte differentiation (Cox et al., 2001) and polarization of the developing oocyte along the anterior/posterior axis (Doerflinger et al., 2010). In the developing nervous system, Baz is essential for the maintenance of stem cell fate in neuroblasts through the establishment of apico-basal polarity, spindle orientation and asymmetric cell division (Kuchinke et al., 1998; Loyer and Januschke, 2020; Schober et al., 1999; Wodarz et al., 1999). In mammalian epithelial cells, Par-3 has been shown to play a crucial role in the assembly and maintenance of tight junctions and adherens junctions as well as epithelial spindle orientation (Chen and Macara, 2005; Hao et al., 2010; Ooshio et al., 2007).

Aside from their cortical localization and roles in regulating cell polarity, several studies have reported that members of the Par complex also localize to the nucleus (Cline and Nelson, 2007; Fang et al., 2007; Perander et al., 2000; Seidl et al., 2012; Speese et al., 2012). Studies in mammalian cell culture have revealed that in response to DNA damage induced by γ-irradiation, Par3 translocates to the nucleus where it associates with DNA-dependent protein kinase (DNA-PK) to mediate DNA double strand break repair (Fang et al., 2007). A study by Speese et al., 2012 revealed that Par proteins also show nuclear localization in *Drosophila* body wall muscles where they promote neuro-muscular junction (NMJ) formation. They reported that Baz localizes to the nuclear envelope, including nuclear envelope foci containing a C-terminal cleavage product of the *Drosophila* Wingless/Wnt1 receptor DFrizzled 2 (DFz2C). At these foci, Baz is required for phosphorylation of the nuclear envelope component LaminC (LamC) by aPKC to promote nuclear envelope budding, facilitating the export of ribonucleoprotein particles containing DFz2C and mRNAs encoding post-synaptic proteins (Speese et al., 2012).

In this study, we sought to further analyze Baz nuclear envelope localization in *Drosophila* to gain insight into any additional functions it may have in the nucleus. By immunostaining analysis and two independent Baz-GFP fusion protein lines (Besson et al., 2015; Buszczak et al., 2007) under the endogenous promotor, we assessed whether Baz nuclear envelope localization was ubiquitous or restricted to specific cell types or developmental stages. Immunostaining analysis using multiple antibodies raised against different domains of Baz revealed that immunostaining for Baz at the nuclear envelope was detectable in some but not all tissues, in particular in polyploid cells. However, to our surprise mutational analysis revealed that Baz nuclear envelope immunostaining persisted in *baz* mutant clones although cortical staining was completely abandoned. Furthermore, analysis of Baz-GFP fusion protein lines showed no Baz-GFP nuclear envelope localization. The same was true for localization of Baz to the NMJ. Thus, our results provide strong evidence that Baz does neither localize to the nuclear envelope nor to the NMJ, requiring a reassessment of its reported function at these subcellular sites.

## RESULTS

### Baz localization at the nuclear envelope is observed with different anti-Baz antibodies

**Fig. 1.**
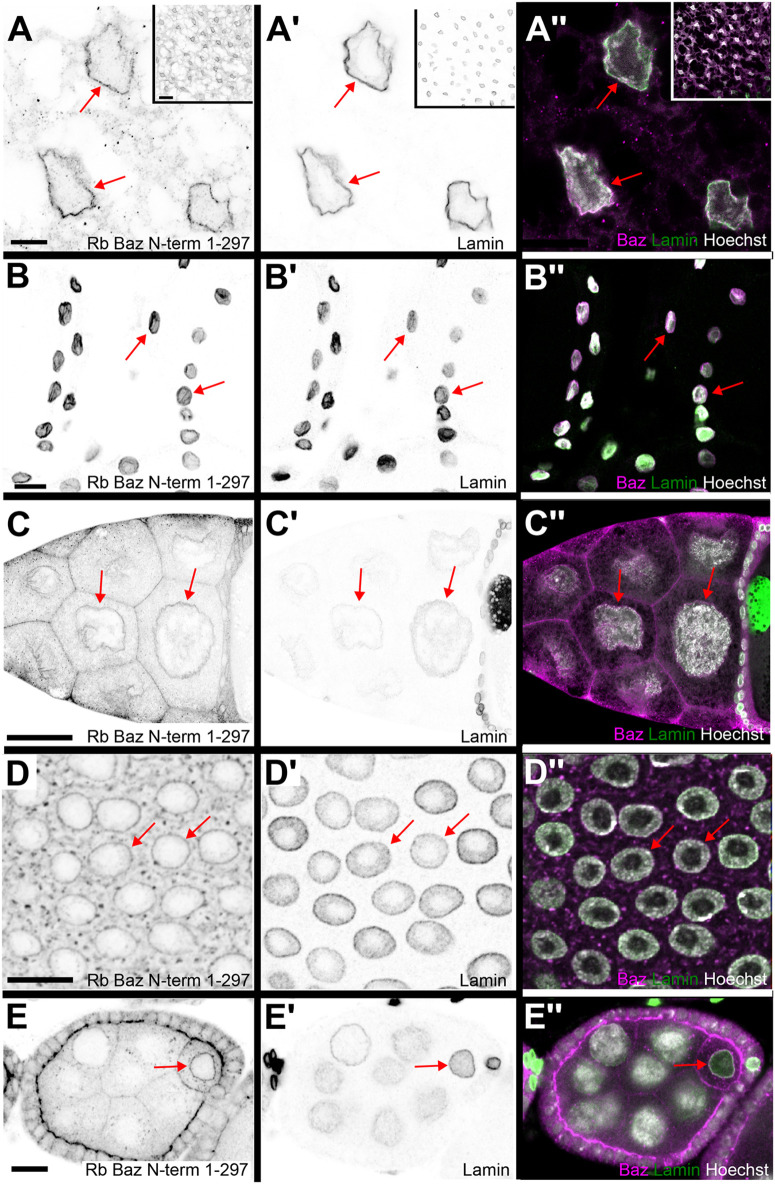
**Bazooka immunostaining colocalizes with Lamin C at the nuclear envelope of several larval and adult tissues.** Fat body tissue (A-A″) and body wall muscles from 3rd instar wild-type larvae (B-B″), as well as adult ovaries (C-E″) were stained with rabbit anti-Bazooka 1-297 (anti Baz, magenta in merge), Lamin C (green in merge) to mark the nuclear envelope and Hoechst (gray in merge) to label DNA. Anti-Baz immunostaining in adipocytes (A) shows a strong signal in the periphery of the nucleus in a pattern very similar to Lamin C (A′). The merged image shows colocalization of both signals (A″). Insets in A-A″ show low magnification overview images of the stained fat body tissue. Body wall muscles show a similar pattern of nuclear envelope localization for anti-Baz staining (B), Lamin C (B′) and their corresponding colocalization (B″). (C-E″) Nuclear envelope localization detected by anti-Baz staining is also observed in nurse cells (C-C″), follicular epithelial cells (D-D″) and oocytes (E-E″) in adult ovaries. Nuclear envelopes are marked by red arrows. Images in the left two columns are displayed in inverted gray scale. Scale bars in A-A″ and E-E″: 10 µm, in B-B″ and D-D″: 20 µm, in C-C″ and insets in A-A″: 50 µm. Genotypes: (A-E) *w^1118^*.

We assessed by indirect immunofluorescence whether Baz also localizes to the nucleus, in addition to its well-described localization to the cytocortex and intercellular junctions. Several antibodies were used to stain fat body tissue dissected from wild-type 3rd instar larvae. Antibodies raised in rabbit and rat against the Baz N-terminal region (amino acids 1-297, numbering refers to isoform PA of Baz; Wodarz et al., 1999, 2000), an antibody raised in guinea pig against the Baz PDZ domains (amino acids 309-747; Shahab et al., 2015) and an antibody raised in guinea pig against a region in the C-terminal half of Baz (amino acids 905-1221, this work) all showed nuclear staining (Fig. S1). For all further analyses we made use of the antibody against the N-terminal region of Baz raised in rabbit, as in the *Drosophila* community it is the most commonly used antibody to detect Baz. Also, it showed the least background staining in comparison to the other antibodies tested. Closer inspection of the immunofluorescence pattern detected by this antibody in fat body showed a localization to the periphery of the nucleus colocalizing with the nuclear envelope component Lamin C (Fig. 1A-A″), indicating that Baz localizes to the nuclear envelope.

### Nuclear envelope immunofluorescence for Baz is observed in different tissues

We next sought to determine whether Baz nuclear envelope localization occurred in a tissue-specific manner. In addition to larval fat body cells (Fig. 1A-A″; Fig. S1), we observed Baz nuclear envelope localization in larval body wall muscle (Fig. 1B-B″), adult ovary nurse cells (Fig. 1C-C″), follicular epithelial cells (Fig. 1D-D″; Fig. S3B) and oocytes (Fig. 1E-E″). Baz nuclear envelope immunostaining signal was absent in embryonic tissue, e.g. epidermis (Fig. S2A-A″) and neuroblasts (Fig. S2A-A″), in larval midgut imaginal islands (Fig. S2B-B″) and in larval wing imaginal disc cells (Fig. S2C-C″).

### Fully functional Baz-GFP fusion proteins do not show nuclear envelope localization

While strong nuclear envelope immunostaining was observed using several independently raised anti Baz antibodies (Fig. 1; Fig. S1), no nuclear envelope localization was detected in follicular epithelial cells and in larval body wall muscles using a Baz-GFP BAC line (Besson et al., 2015) (Figs S3C-D″, S4A,A') nor in a GFP-Baz protein-trap line (Buszczak et al., 2007) (Figs S3E-F″, S4C,C'). In the GFP-Baz protein-trap line an engineered exon encoding for GFP is inserted into the second untranslated exon (Fig. S5). This exon encoding for GFP is predicted to be spliced in frame into the mRNAs RA and RC encoding for isoforms PA and PC whose translation starts in exon 1 (Fig. S5), resulting in insertion of GFP between amino acid residues K40 and P41 of isoforms PA and PC. The transcripts RB and RD encoding Baz isoforms PB and PD have their translation start within exon 3 and thus cannot form fusion proteins with GFP inserted in exon 2 (Fig. S5). However, GFP-Baz protein trap flies are homozygous viable and are phenotypically indistinguishable from wild-type flies, indicating that the corresponding GFP fusion protein is fully functional and faithfully reflects the expression pattern and subcellular localization of Baz isoforms PA and PC. The BAC line integrates the GFP within exon 10 between amino acid residues L1424 and Q1425 of isoform PA, giving rise to GFP fusion proteins for all four isoforms (Fig. S6) (Besson et al., 2015). Like the protein-trap GFP-Baz fusion protein, the Baz-GFP fusion protein in the BAC line is fully functional as it completely rescued lethality and fertility of the *baz^EH747^* (Fig. S7D-D″) and *baz^815-8^* alleles (Besson et al., 2015). For both lines GFP expression was detected at adherens junctions, colocalizing with the signal from the Baz N-terminal antibody (Fig. 3E,G; Figs S3C,C',E,E', S7D',D″,E',E″). However, even after signal enhancement using an anti-GFP antibody we did not detect a signal at the nuclear envelope in follicular epithelial cells like seen when using the anti-Baz antibodies (Fig. S3D',D″,F',F″), nor at the nuclear envelope of the oocyte (Fig. S7D',D″,E',E″).

### Baz nuclear envelope immunostaining in the ovary is an artifact

**Fig. 2.**
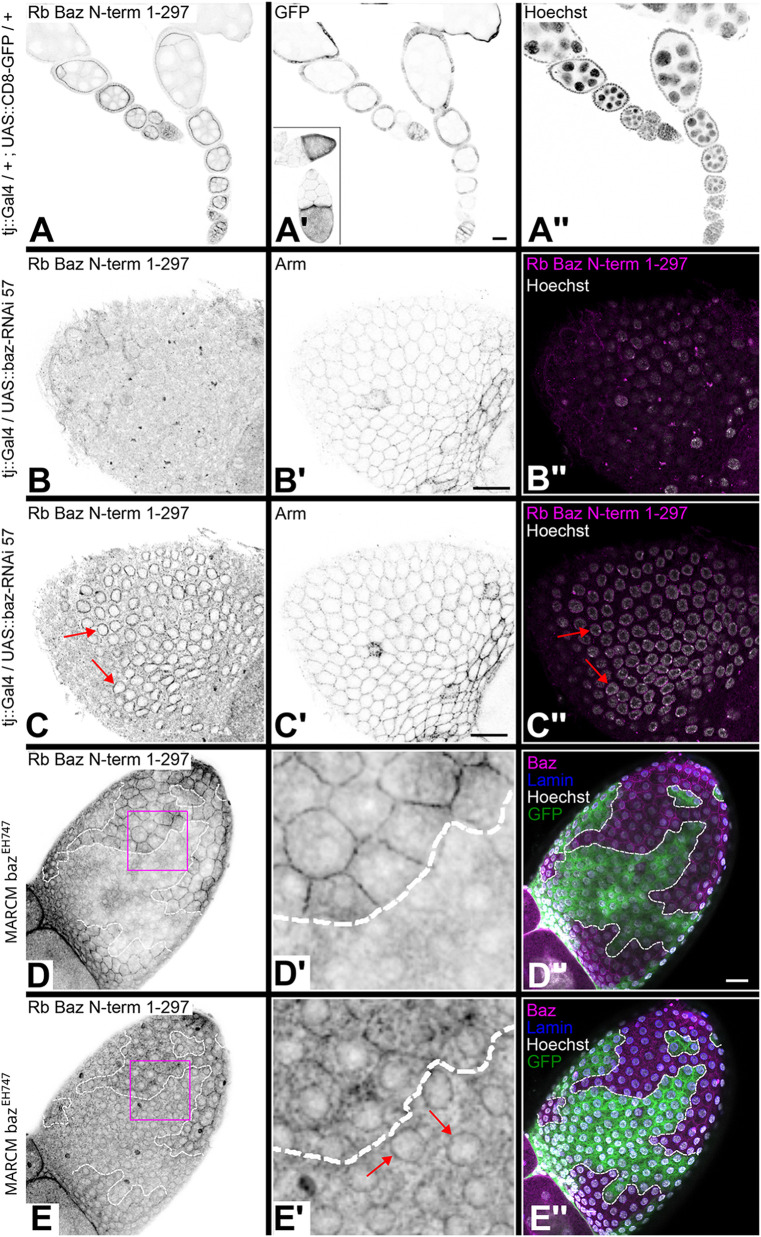
**Nuclear envelope localization of Baz in follicular epithelial cells is a staining artifact.** (A-A″) Expression pattern of the tj::Gal4 driver line in follicular epithelial cells. Ovarioles expressing mCD8-GFP under control of tj::Gal4 were stained with rabbit anti-Bazooka 1-297 (A), GFP (A′) and Hoechst (A″). Anti-Baz staining in early egg chambers shows an apical junctional signal in follicular epithelial cells (A). UAS::CD8-GFP under control of the tj-Gal4 driver shows GFP in all follicular epithelial cells from early stages on (A′). At later stages GFP is expressed homogenously in follicle cells covering the oocyte (inset in A′). (B-C″) baz-RNAi driven by tj::Gal4 leads to strong reduction of anti-Baz staining at adherens junctions in the follicular epithelium (B) whereas junctional β-catenin (Arm) staining is clearly visible (B′). The merged image of anti-Baz staining (magenta) and Hoechst DNA stain (gray) is shown in B″. C-C″ shows the same egg chamber as in B-B″ imaged at the level of the nuclei. Anti-Baz staining is still detectable at the nuclear envelope (C, red arrows) although there is no staining detectable at the junctions (compare to B and to Arm staining shown in B′, C′). The merged image of anti-Baz staining (magenta) and Hoechst DNA stain (gray) is shown in C″. Confocal sections shown in B and C are 1.45 µm apart. (D-E″) MARCM loss-of-function clones for the null allele *baz^EH747^* in the follicular epithelium imaged at two different focal planes. (D-D″) At the focal plane showing the adherens junctions, *baz^EH747^* homozygous mutant cells marked by GFP (green in D″) show a complete loss of junctional staining with the anti-Baz antibody (D,D′). (E-E″) In the focal plane of the nuclei, anti-Baz staining is still detectable at the nuclear envelope in *baz^EH747^* homozygous mutant cells marked by GFP (green in E″) although these cells have lost junctional staining for Baz (red arrows in E′). Images in the left two columns are displayed in inverted gray scale. Merged images in D″, E″ show Baz (magenta), Lamin C (blue), GFP (green) and Hoechst (gray). Clone borders in D-E″ are marked by a white dotted line. Close-ups of the areas marked in D, E with a magenta square are shown in D′, E′. Confocal sections shown in D and E are 2.7 µm apart. Scale bars: 20 µm. Genotypes: (A) tj::Gal4/+ ; UAS::mCD8-GFP/+. (B-C) tj::Gal4/UAS::baz-RNAi 57. (D-E) *baz^EH747^* FRT19A/ *tubP::Gal80LL1 hsFLP FRT19A; tubP::Gal4 UAS::mCD8-GFP*.

In the follicular epithelium, Baz immunostaining detected Baz at the adherens junctions (Fig. S3A) and in a different focal plane at the nuclear envelope (Fig. S3B). To test whether both junctional and nuclear envelope staining of Baz was specific, we eliminated *baz* expression in the follicular epithelium using two different methods. First, we used RNAi against *baz* driven by *traffic jam* (*tj*)::Gal4, which is expressed in all follicle cells of the developing egg chamber until stage 12 (Fig. 2A') (Li et al., 2003). During early stages of egg chamber development the expression of *tj::*Gal4 in the follicle cells is heterogeneous (Fig. 2A'), while it becomes homogeneous in older egg chambers (inset in Fig. 2A'). Whereas the junctional staining for Baz close to the apical surface of the follicular epithelium was lost upon RNAi against *baz* (Fig. 2B), the staining at the nuclear envelope persisted in the same egg chamber imaged at the optical plane containing the nuclei (Fig. 2C). The staining for Armadillo/beta-catenin (Arm) (Fig. 2B',C') shows junctions that are still intact although Baz is downregulated (Shahab et al., 2015).

For clonal analysis the strong loss-of-function allele *baz^EH747^* was used, where a point mutation in exon 4 results in a premature stop close to the N-terminus of all four isoforms (the codon for amino acid residue Q51 is mutated to a stop in isoform A) (Krahn et al., 2010). In follicular epithelial cells loss of Baz has no relevance for the integrity of adherens junctions and development of the egg chamber is unaffected (Shahab et al., 2015). FLP-FRT and MARCM clones were generated in follicle cells and in the germline. Both small and large clones in the follicle epithelium showed the loss of Baz staining at the junctions (Fig. 2D,D') but a persistent immunostaining signal for Baz around the nucleus (Fig. 2E,E'). Large follicle cell clones encompassing all follicle cells of an egg chamber marked by the loss of nuclear GFP (Fig. S7B') showed the loss of junctional Baz in the *baz^EH747^* mutant follicle cells (Fig. S7B″). The loss of Baz in the follicle cells did not result in any apparent junctional defect and the junctional marker Arm was still localized as in wild type (Fig. S7B‴). In the germ line Baz was detectable at the junctions between the germ line cells, in particular between nurse cells and the oocyte. In addition, a strong signal was consistently detected at the nuclear envelope of the oocyte (Fig. S7A″). While in *baz^EH747^* mutant germ line clones marked by the loss of nuclear GFP (Fig. S7C′), the junctional staining within the germline was lost, the staining around the oocyte nucleus persisted (Fig. S7C″). The staining for Arm revealed that the junctions between the germline cells were still intact (Fig. S7C‴). The staining around the oocyte nucleus was not detectable with the anti GFP antibody in the Baz-GFP BAC line (Fig. S7D′) nor in the GFP-Baz protein-trap line (Fig. S7E′). Staining with rabbit anti Baz antibody again showed the oocyte nuclear envelope marked in these egg chambers (Fig. S7D″,E″).

### Baz nuclear envelope and NMJ immunostaining in the L3 body wall muscle does not reflect the true Baz localization

**Fig. 3.**
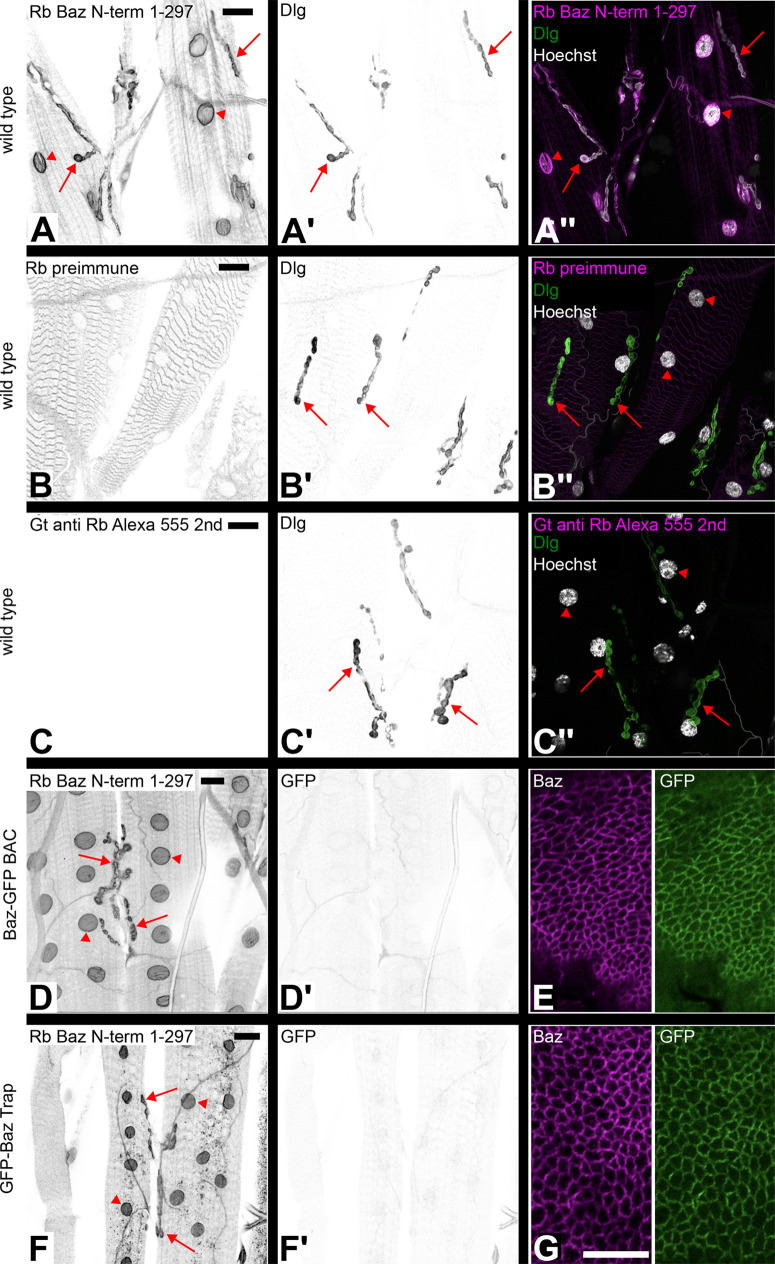
**In larval body wall muscles staining of the nuclear envelope and the NMJ using the anti-Baz antibody is not reflected by the subcellular localization of Baz-GFP in the BAC line and GFP-Baz in the gene trap line.** (A-A″) Larval body wall muscles stained with anti-Baz antibody (A) and Dlg antibody (A′) to mark the neuromuscular junctions (NMJ). Anti-Baz staining marks the nuclear envelope (red arrowheads) and the NMJs (red arrows, A). The merged image is shown in A″. (B-B″) Antibody staining with the pre-immune serum of the rabbit in which the anti-Baz antibody had been raised (B). Neither the nuclear envelope nor the NMJs are stained (B). Dlg staining of the NMJ is unaffected (red arrows, B′). The merged image is shown in B″. (C-C″) As a negative control, staining without primary rabbit antibody was performed, resulting in no signal (C). Dlg staining of the NMJ is unaffected (red arrows, C′). The merged image is shown in C″. (D,E) Antibody staining of body wall muscle (D,D′) and wing imaginal disc (E) of a larva carrying the Baz-GFP BAC construct. Anti-Baz staining shows a signal at the nuclear envelope (red arrowheads, D) and the NMJ (red arrows, D). Anti GFP staining shows only background signal in the body wall muscle (D′). Staining of a wing imaginal disc of the same larva as in D shows colocalization of Baz (magenta) and GFP (green) signals at epithelial junctions (E). Antibody staining of body wall muscle (F,F′) and wing imaginal disc (G) of a larva of the GFP-Baz protein-trap line. Anti-Baz staining shows a signal at the nuclear envelope (red arrowheads) and the NMJ (red arrows, F). Anti GFP staining shows only background signal in the body wall muscle (F′). Staining of a wing imaginal disc of the same larva as in F shows colocalization of Baz (magenta) and GFP (green) signals at epithelial junctions (G). Images in the left two columns are displayed in inverted gray scale. Scale bar in A: 20 µm, valid for panels (A-D′, F, F′). Scale bar in G: 10 µm, valid for E, G. Genotypes: (A-C) *w^1118^*. (D, E) *w, P{CaryP, PB[BAC BazsfGFP2]attP18}* (on X). (F, G) *w, baz-GFP^CC01941^*.

In parallel to our observations in the ovary we looked for Baz nuclear localization in the larval body wall muscle. Baz immunostaining was detectable at the nuclear envelope and at the neuromuscular junction (NMJ) as published before (Ruiz-Canada et al., 2004; Speese et al., 2012) (Fig. 3A-A″). To test if this signal was specific for the antibody, we stained with the pre-immune serum, revealing a striped pattern in the muscle, but no signal at the NMJ or the nucleus (Fig. 3B-B″). Staining with the secondary antibody only without any primary antibody resulted in no signal at the NMJ or the nucleus (Fig. 3C-C″). Anti Discs large antibody and Hoechst were used in the same stainings as control to detect the NMJs and the nuclei (Fig. 3A-C″). Together, these experiments showed that the Baz antibody was indeed responsible for the signal at the NMJ and the nuclear envelope of somatic body wall muscles.

By contrast, we did not detect GFP at the NMJ or at the nuclear envelope in larval body wall muscles in the Baz-GFP BAC and the GFP-Baz trap lines stained with anti GFP antibody (Fig. 3D′,F′), whereas we observed a strong signal with the anti Baz antibody in these lines (Fig. 3D,F). In the wing imaginal discs of the same L3 larvae we observed colocalization of Baz and GFP immunostaining at intercellular junctions as expected (Fig. 3E,G). These findings were confirmed by analysis of fixed larval tissues that were imaged for GFP fluorescence without anti GFP antibody staining (Fig. S4). Neither in the Baz-GFP BAC line (Fig. S4A,A′), nor in the GFP-Baz trap line (Fig. S4C, C′) any nuclear envelope or NMJ signal was detectable in somatic muscles, whereas junctional signal in wing imaginal discs was readily detectable in both lines (Fig. S4B,D).

It has been published that heterozygous *baz^4^* mutant larvae show a significant decrease in immunofluorescence signal of Baz and also of Spectrin at the NMJ (Ruiz-Canada et al., 2004). Another publication showed a significant decrease in Baz and Spectrin immunostaining at the NMJ of larvae heterozygous for the *baz^815-8^* allele (Ramachandran et al., 2009). We did not attempt to reproduce these findings. However, in our hands mitotic clones generated with FRT chromosomes carrying these latter two *baz* alleles showed polarity phenotypes in the follicular epithelium, whereas clones of the clean *baz^EH747^* null allele did not show any polarity defect (Shahab et al., 2015), raising the possibility that the NMJ phenotypes observed by Ruiz-Canada et al. (2004) and Ramachandran et al. (2009) were caused by second site mutations on these chromosomes rather than by reduced Baz activity.

**Fig. 4.**
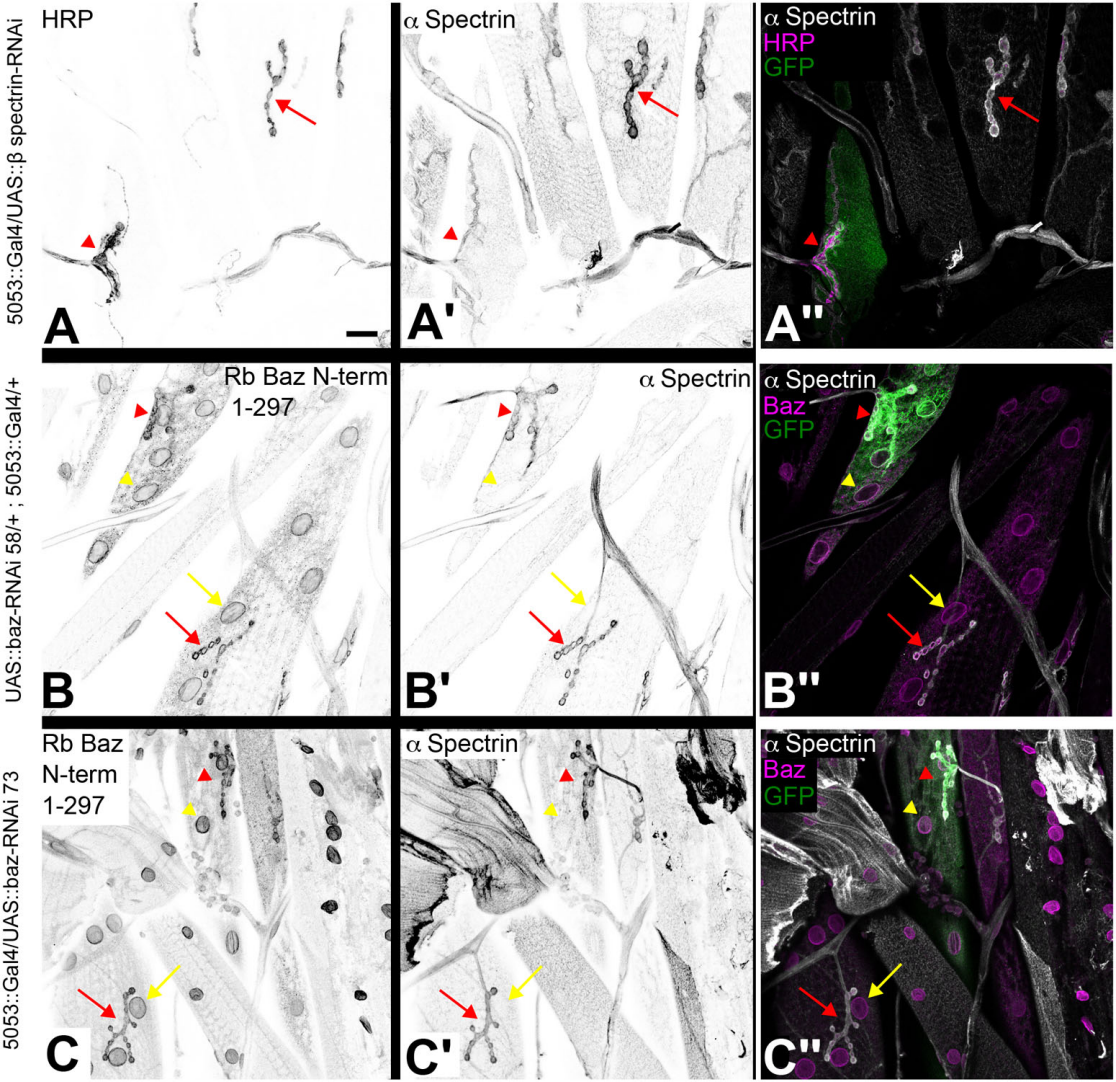
**Nuclear envelope and NMJ localization of Baz in larval body wall muscles is a staining artifact.** (A-A″) β-Spectrin-RNAi was driven in muscle M12 by the 5053::Gal4 line. Larval body wall muscles were stained with antibodies against HRP (A), α-spectrin (A′) and GFP (green in merged image A″). Co-expression of GFP marks muscle M12 (red arrowhead), where the β-Spectrin-RNAi construct is driven (A″). The red arrow points to a control muscle (GFP-negative) not expressing β-Spectrin-RNAi to compare the α-spectrin signal at NMJs. (A′,A″) RNAi against β-Spectrin results in downregulation of α-Spectrin in the NMJ of muscle M12 (red arrowhead, A′). In the neighboring control muscle, strong α-Spectrin staining is detectable at the NMJ (red arrow, A′). The merged image with GFP (green), α-Spectrin (gray) and HRP (magenta) is shown in A″. (B-C″) Baz-RNAi with two different constructs [baz-RNAi 58 (B-B″) and baz-RNAi 73 (C-C″)] does not show downregulation of anti-Baz staining at the nuclear envelope (yellow arrowhead, B, C) or the NMJ (red arrowhead, B, C) in M12 in comparison to a control muscle not expressing baz-RNAi (yellow and red arrow, B, C). α-Spectrin is also not downregulated in the NMJ of muscle M12 (red arrowhead, B', C') in comparison to a control muscle (red arrow, B′, C′). Merged images with GFP (green), α-Spectrin (gray) and Baz (magenta) are shown in B″, C″. Images in the left two columns are displayed in inverted gray scale. Scale bar in A: 20 µm, valid for all panels. Genotypes: (A) *5053::Gal4 M12/UAS::β-spectrin-RNAi* (on III.). (B) *UAS::baz-RNAi 58/+ ; 5053::Gal4 M12/+*. (C) *5053::Gal4 M12/UAS::baz-RNAi 73* (on III.).

To reassess a potential localization of Baz at NMJs, we downregulated Baz by RNAi. Clonal analysis using a null allele of *baz* is not feasible in the muscle due to the syncytial nature of this tissue. To test whether RNAi works in body wall muscles we conducted *β-spectrin*-RNAi using the muscle specific (M12) driver line 5053-Gal4. We observed a strong decrease of α-spectrin staining in the postsynaptic membrane within the M12 muscle as published before (Pielage et al., 2006) (Fig. 4A-A″). We pursued the same approach to downregulate Baz in muscle M12. The functionality of *baz*-RNAi was demonstrated in the follicular epithelium, where expression under the control of tj::Gal4 led to strong downregulation of Baz at epithelial junctions (Fig. 2B). While it has been proposed that Baz is localized at the postsynaptic membrane within the NMJ (Ruiz-Canada et al., 2004), we did not detect any decrease in immunoreactivity for Baz or α-Spectrin upon *baz*-RNAi in M12 compared to other muscle segments not expressing *baz*-RNAi (Fig. 4B-C″; compare NMJs marked by red arrowhead and red arrow). Baz nuclear envelope localization remained unaffected as well. We also did not see any downregulation of Baz or α-spectrin upon *baz*-RNAi in M12 at 29°C, when the UAS-Gal4 system is maximally active (Fig. S8). Taken together, our data strongly indicate that the nuclear envelope and NMJ immunostaining pattern observed using anti-Baz antibodies does not reflect the true Baz localization.

## DISCUSSION

### Baz nuclear envelope localization is an artifact of immunostaining

In line with results from (Speese et al., 2012), our initial immunostaining analysis revealed strong localization of Baz to the nuclear envelope and colocalization with the nuclear envelope protein Lamin C. The fact that four different anti Baz antibodies raised against three different regions of the Baz protein showed nuclear localization provided strong evidence to believe that the observed immunostaining pattern was unlikely to be an immunostaining artifact. Further analysis revealed that localization of Baz to the nuclear envelope was detectable only in specific cell types. We found Baz nuclear envelope immunostaining mostly in polyploid cells undergoing endoreduplication such as larval fat body cells, larval body wall muscle, nurse cells and late-stage follicular epithelial cells, but also in diploid oocytes.

However, analysis of Baz immunostaining in mutant clones for the strong loss-of-function allele *baz^EH747^* lacking all but the first 51 amino acid residues of Baz indicated that the observed nuclear envelope localization pattern was not specific to Baz. We initially speculated whether the persistence of Baz nuclear envelope immunostaining in the *baz* mutant follicular epithelial cells and oocyte could be due to increased Baz protein stability and low turnover of Baz associated with the nuclear envelope. However, this is unlikely given that no change in the intensity of Baz nuclear envelope staining was observed when large late-stage follicular epithelial clones were compared to small early-stage clones. If perdurance would be the explanation for the persistent nuclear envelope staining, then the signal should become weaker as cells divide and the tissue expands. Similarly, in *baz* mutant nurse cells, Baz staining at the junctions was completely lost whereas no change in nuclear envelope staining intensity was observed.

We also considered the possibility that the short N-terminal peptide of 51 amino acids that may still be expressed in the *baz^EH747^* allele could give rise to the observed staining at the nuclear envelope of follicular epithelial cells. However, we consider this possibility very unlikely for two reasons: 1) RNAi affects the *baz* mRNA and thus should knock down all epitopes to the same degree. However, we see a complete loss of junctional Baz signal but no reduction of the signal at the nuclear envelope or the NMJ upon RNAi targeting *baz*. 2) The GFP-Baz fusion proteins do not show any signal at the nuclear envelope upon imaging of the native GFP fluorescence or upon antibody staining with an anti GFP antibody, although both the Baz-GFP BAC line and the GFP-Baz protein trap line express full-length Baz including the N-terminal epitope that is potentially still expressed in the *baz^EH747^* allele.

Thus, our data strongly indicate that Baz does not localize to the nuclear envelope and that nuclear envelope immunostaining observed with anti-Baz antibodies is due to cross-reactivity with an unidentified epitope. We can only speculate about the reason for the nuclear envelope signal detected with the different anti Baz antisera. All four sera were raised against GST fusion proteins, so it could be that antibodies against GST that are present in the antisera cross-react with a component of the nuclear envelope. However, as we were interested in a potential function of Baz at the nuclear envelope, we did not further investigate this after we found that signal to be unrelated to Baz.

### Baz does not localize to the postsynaptic membrane of the neuromuscular junction

Finally, our data also revealed that the published localization of Baz to the postsynaptic membrane of the NMJ (Ramachandran et al., 2009; Ruiz-Canada et al., 2004; Speese et al., 2012) is an artifact, as the staining is unaffected by RNAi against Baz and neither the GFP-Baz trap line nor the Baz-GFP BAC line showed any GFP signal at the NMJ. Work from the Budnik lab reported NMJ phenotypes such as reduced staining intensity for α-Spectrin and reduced number of synaptic boutons in animals heterozygous mutant for the *baz* alleles *baz^4^* and *baz^815-8^* as well as upon RNAi against *baz* (Ramachandran et al., 2009; Ruiz-Canada et al., 2004). We did not attempt to reproduce these findings because our data do not provide evidence for localization of Baz at the NMJ. The reported effects on the NMJ may be explained by the existence of second site mutations on the chromosomes carrying the *baz^4^* and *baz^815-8^* alleles used for the analyses. These second site mutations apparently also cause defects in epithelial apical-basal polarity that are not observed using clean null alleles of *baz* (Shahab et al., 2015).

Altogether, the reported findings for a function of Baz at the nuclear envelope (Speese et al., 2012) and at the NMJ (Ramachandran et al., 2009; Ruiz-Canada et al., 2004) should be regarded with great caution as we did not find any evidence for localization of Baz to these subcellular structures.

## MATERIALS AND METHODS

### Fly stocks and genetics

The following fly stocks were used in this study: *w^1118^* (was used as wild-type control, BL 3605), Baz-GFP BAC (Besson et al., 2015), GFP-Baz gene trap CC01941 (BL 51572) (Buszczak et al., 2007) *UAS::baz-RNAi57* (VDRC v2915, on II.), *UAS::baz-RNAi58* (VDRC v2914, on II.), *UAS::baz-RNAi73 HMS01412* (BL 35002), *UAS::β-spectrin-RNAi* (Pielage et al., 2005), *UAS::CD8-GFP* (BL 32184), *traffic jam*::Gal4 (Li et al., 2003), *5053::Gal4 M12* (BL 2702), *FRT19A* (BL 1709), *baz^EH747^ FRT19A*, (Shahab et al., 2015), *hsFlp^122^ FRT19A H2AvD-GFP* (BL 32045), *FRT19A tubP::Gal80LL1 hsFLP; tubP::Gal4 UAS::mCD8-GFP* (for generation of MARCM clones, gift from Heinrich Reichert). Stock numbers from the Bloomington *Drosophila* Stock Center (BL #) and from the Vienna *Drosophila* Research Center (VDRC #) are given in parentheses. Crossings for RNAi experiments were set up at 25°C if not indicated otherwise. For generating follicle cell clones in ovaries by Flipase-mediated mitotic recombination of the FRT sites flies were heat shocked for 1 h at 37°C 5-7 days prior to preparation of the ovaries. For generation of germ line clones by Flipase-mediated mitotic recombination of the FRT sites flies were heat shocked twice for 2 h at 37°C on two consecutive days in late 2nd, early 3rd instar larval stages.

### Preparation of ovaries

One day before preparation the females were fed with fresh yeast. Ovaries were removed and ovariole tubules separated by pipetting the ovaries prior to fixation two times with a cut 1000 µl tip to enlarge tip opening. Ovaries were fixed in 3.7% formaldehyde, washed three times in PBS and blocked in PTX (0,1% Trixon X-100 in PBS) for at least 3 h.

### Preparation of larval muscle

L3 larvae were washed in PBS to remove residual food and placed on a plate with PBS. Larvae were fixed with needles and cut open between the lateral trunks of the tracheae along the a/p-centerline. Larval cuticle was nicked at the anterior and posterior end to the lateral side and the cuticle was unfolded to fix it with needles onto the plate to flatten it. Inner organs were removed, imaginal discs remained. Torsos were washed with PBS to remove residual tissue and then shortly once with 3.7% formaldehyde in PBS and then fixed for 15 min in 3.7% FA. Fixative was removed and the torso washed three times with PBS. After the needles were removed, the torsos were transferred to a tube and washed three times with PTX (0.1% Triton X-100 in PBS) for 10 min.

### Immunostainings, antibodies and imaging

The following antibodies were used for immunostainings: rabbit anti Baz-N-term (aa 1-297) (Wodarz et al., 2000) 1:1000, rabbit anti Baz-N-term (aa1-297) pre-immune 1:1000, rat anti Baz-N-term (aa 1-297) (Wodarz et al., 1999) guinea pig anti Baz-PDZ (aa 309-747) (Shahab et al., 2015) 1:1000, guinea pig anti Baz-C-term (aa 905-1221) 1:1000 (this work), mouse anti α-Spectrin (3A9, DSHB) 1:10, mouse anti Dlg (4F3, DSHB) 1:20, mouse anti Armadillo (N2-7A1, DSHB) 1:20, mouse anti Lamin C (ADL 76.10, DSHB) 1:100, rat anti DE-Cadherin (DCAD2, DSHB) 1:5, goat anti HRP-Alexa647 (Jackson ImmunoResearch), mouse anti GFP (A11120 Molecular Probes) 1:1000, rabbit anti GFP (A11122 Molecular Probes) 1:1000. Secondary antibodies conjugated to Alexa-Fluor-488/555/647 (Invitrogen) were used at 1:400. DNA was stained with HOECHST 33258 (Sigma). Immunostainings for the 1st and 2nd antibodies were performed in 5% NHS in PTX. Tissues were imaged on a Zeiss LSM880 Airyscan confocal microscope using 25x LCI Plan Neofluar NA 0.8 and 63x Plan Apochromat NA 1.4 oil immersion objectives. If not stated otherwise in the figure legend, all confocal images are single optical sections taken at a pinhole setting of 1 Airy unit. Images were processed with Zen black software (Zeiss) without contrast enhancement. Figures were assembled with Inkscape 1.2 (Inkscape.org) and Powerpoint (Microsoft).

### Image analysis and statistics

Images were analyzed for the presence or absence of a fluorescence signal at the nuclear envelope or the NMJ compared to negative or positive controls, either in the same tissue (mutant clones in the follicular epithelium, RNAi in a specific body wall muscle, junctional versus nuclear signal, anti-Baz staining versus Baz-GFP signal) or in samples processed in parallel (ovaries with follicle cell and germ line clones). Fluorescence intensities were not quantified because the results were obvious and fully penetrant. Therefore, no statistical analysis of the results was required.

## Supplementary Material

10.1242/biolopen.060544_sup1 Supplementary information
