# Supplementary material for: Re-assessment of the subcellular localization of Bazooka/Par-3 in Drosophila: no evidence for localization to the nucleus and the neuromuscular junction
Source: Biol Open. 2024 Jun 27;13(6):bio060544. doi: 10.1242/bio.060544 (PMC11225583; doi:10.1242/bio.060544)
Supplement: Supplementary information [file biolopen-13-060544-s1.pdf]

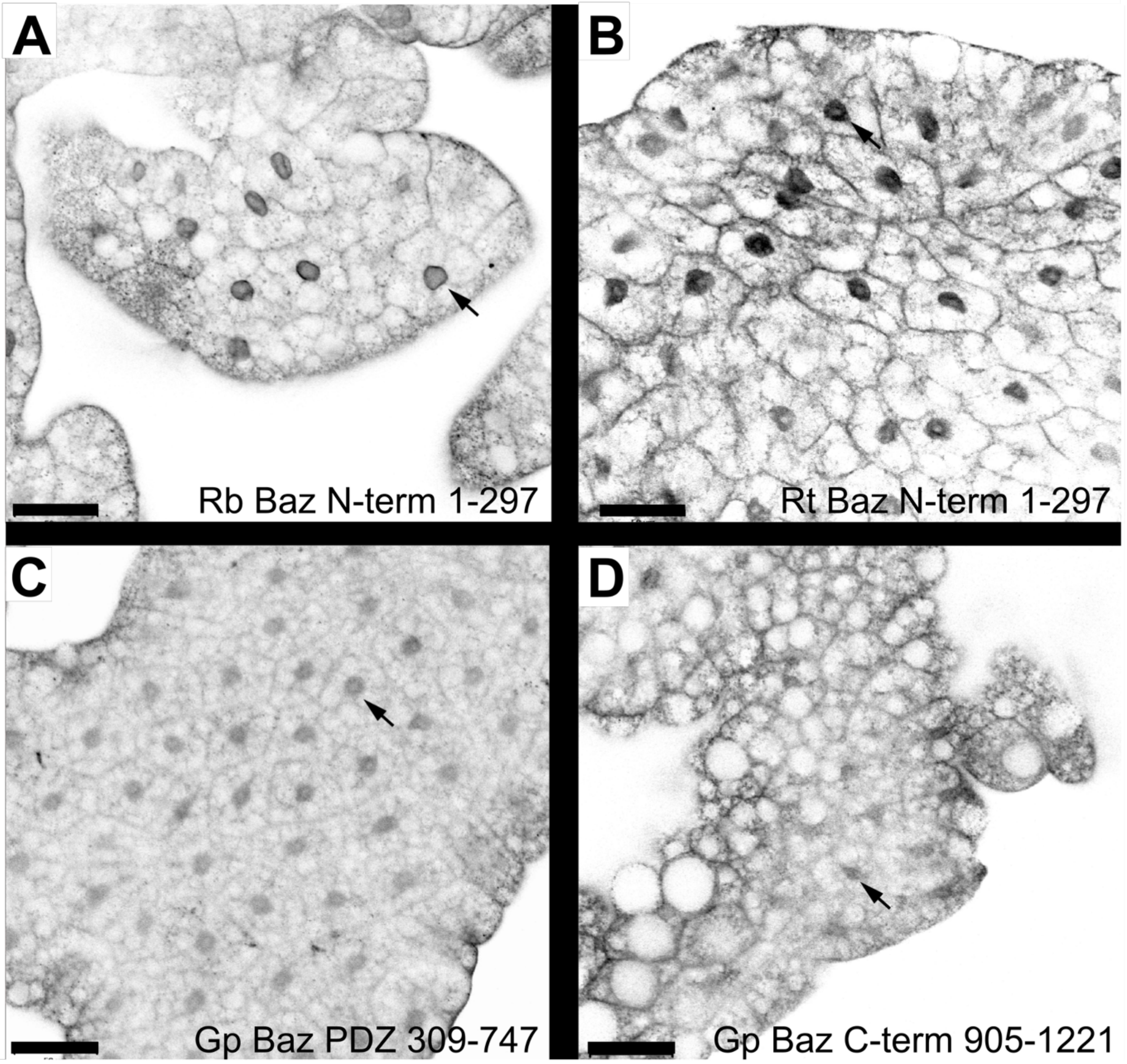


**Fig. S1. Baz nuclear envelope localization is observed with multiple antibodies raised against non-overlapping domains of Baz.** (A-D) Fat body tissue dissected from wild type 3rd instar larvae and stained with different anti-Baz antibodies. Rabbit anti-Baz N-term 1-297 (A) and Rat anti-Baz N-term 1-297 (B) were raised against a GST fusion protein containing amino acids 1-297 from the N-terminal region of Baz. Guinea pig anti-Baz PDZ 291-737 (C) was raised against a GST fusion protein containing amino acids 291-737 of Baz corresponding to the PDZ domains. Guinea pig anti-Baz C-term 905-1221 (D) was raised against a GST fusion protein containing amino acids 905-1221 of Baz. Images are displayed in inverted gray scale. Arrows indicate nuclei. Scale bars = 50 µm. Genotypes: (A-D) $w^{1118}$.

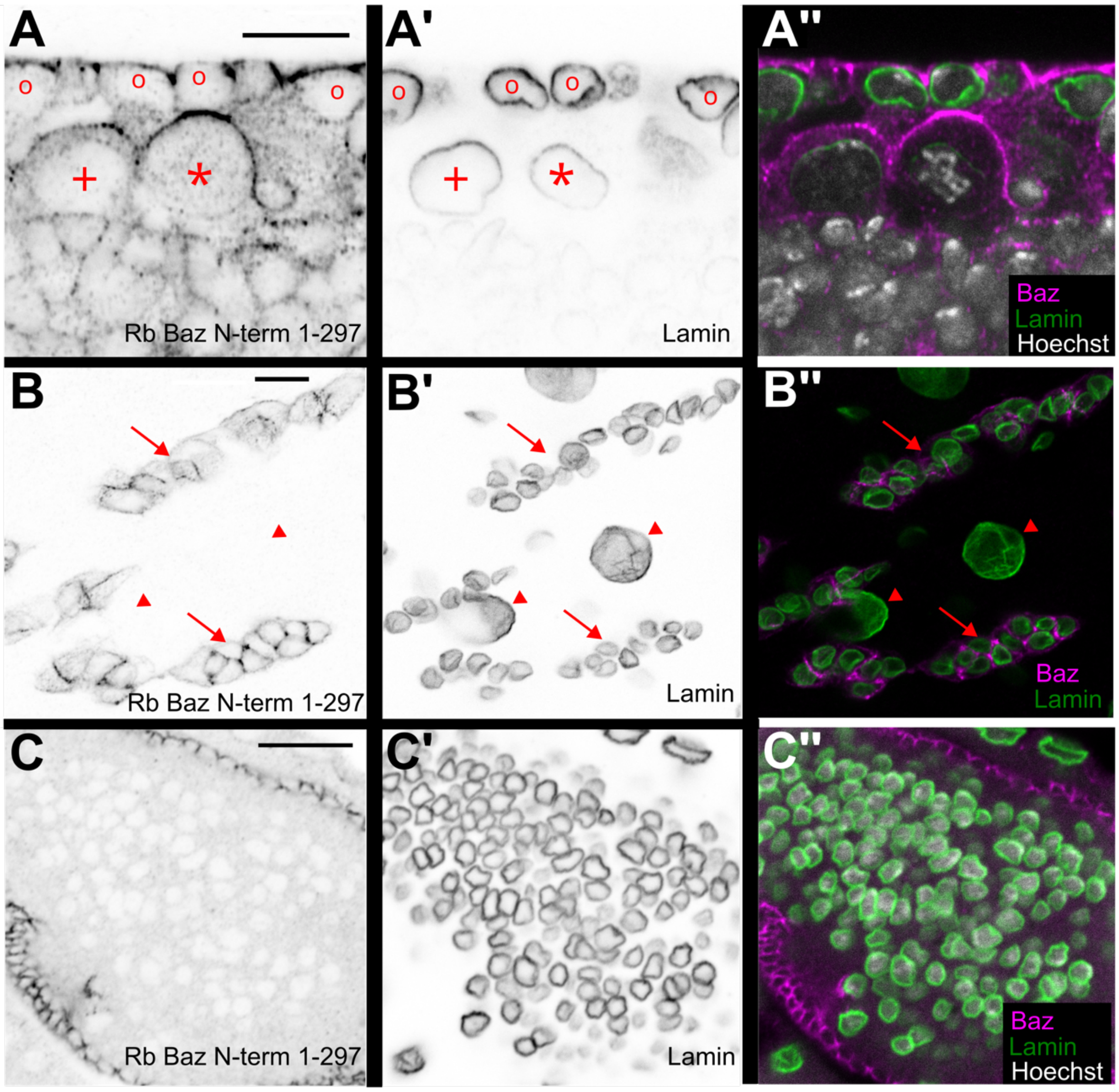


**Fig. S2. Anti-Baz staining is not detectable at the nuclear envelope of embryonic epidermal cells and neuroblasts, larval midgut imaginal islands and imaginal disc cells.** (A-A'') Sagittal optical section of a stage 8 embryo stained with anti-Baz (A, magenta in [A'']), Lamin C (A', green in [A'']) and Hoechst (gray scale in [A'']). An interphase neuroblast is marked by (+) and a prophase neuroblast is marked by (*). Epidermal epithelial cells apical to the neuroblasts are marked by (o). Anti-Baz staining marks the apical adherens junctions of the epidermis and the apical cortex of neuroblasts, but is absent from the nuclear envelope (A, A''). (B-B'') a 3$^{rd}$ instar larval midgut was stained for Baz (B, magenta in B'') and Lamin C (B', green in B''). Baz is expressed in midgut imaginal islands (arrows) but absent in polyploid enterocytes (arrowheads) and localizes to the cortex, but not to the nuclear envelope labeled by Lamin. (C-C'') Detail of a third instar wing imaginal disc stained with anti-Baz (C, magenta in [C'']), Lamin C (C', green in [C'']) and Hoechst (gray in [C'']). The optical section shown is at the focal plane of the nuclei in the center and at the plane of the adherens junctions at the margins of the wing disc. Baz staining is strongly detectable at adherens junctions, but not at the nuclear envelope. Images in the left two columns are displayed in inverted gray scale. Scale bars = 10 µm. Genotypes: (A-C) *w*$^{1118}$.

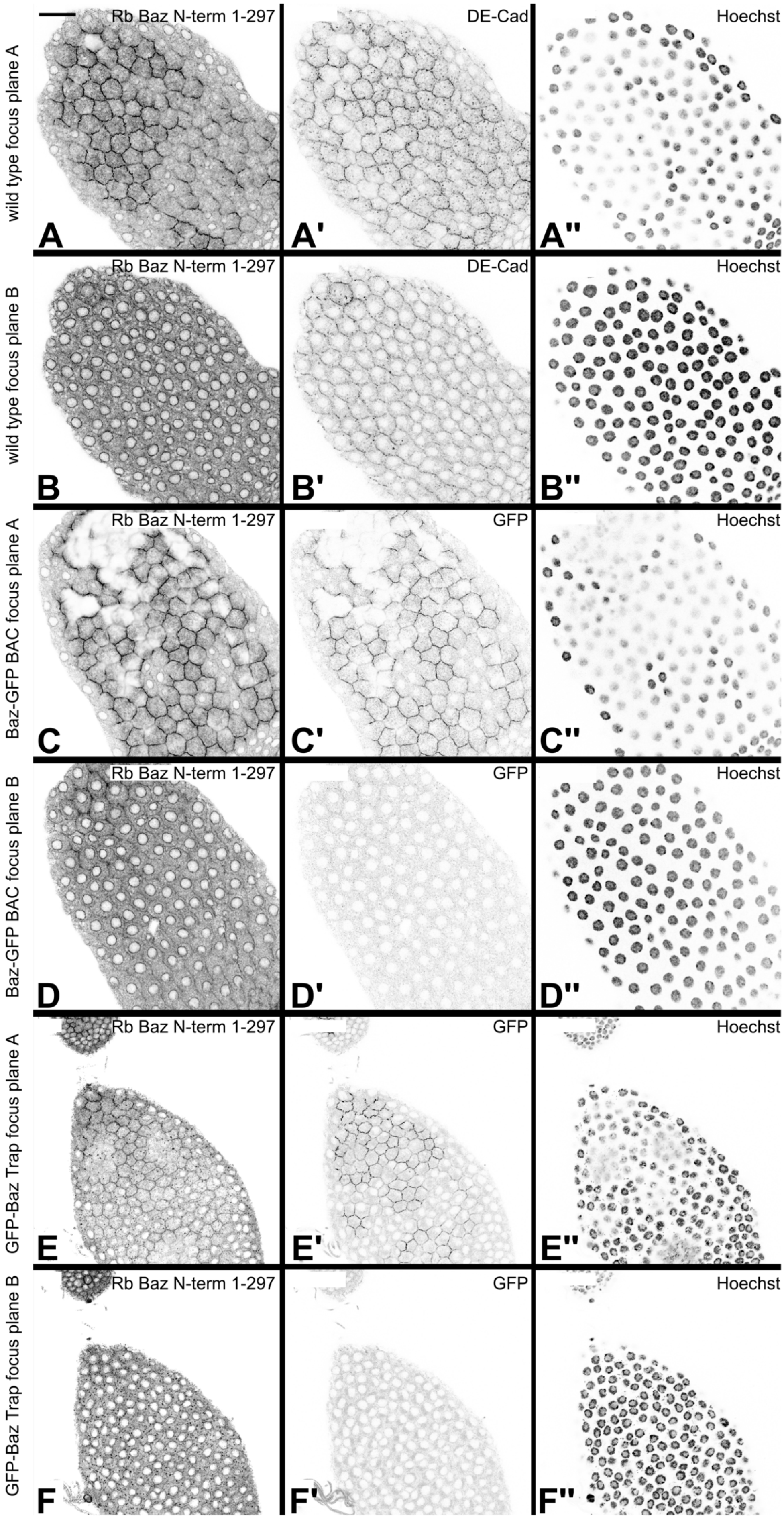
wild type focus plane A
wild type focus plane B
Baz-GFP BAC focus plane A
Baz-GFP BAC focus plane B
GFP-Baz Trap focus plane A
GFP-Baz Trap focus plane B
Rb Baz N-term 1-297
DE-Cad
GFP
Hoechst
A
A'
A''
B
B'
B''
C
C'
C''
D
D'
D''
E
E'
E''
F
F'
F''

**Fig. S3. Anti-Baz staining colocalizes with Baz-GFP and GFP-Baz in epithelial junctions of the follicular epithelium but not at the nuclear envelope.** (A-B'') Follicular epithelium of a wild type egg chamber imaged at the focal plane of the adherens junctions (A-A'') and the nuclei (B-B'') stained with anti-Baz (A, B), DE-Cad (A', B') and Hoechst (A'', B''). Junctional staining of Baz colocalizes with DE-Cad (A, A') whereas the nuclear envelope staining of Baz does not show any overlap with DE-Cad staining (B, B'). Hoechst staining in (A'', B'') is shown to demonstrate the focal plane of the respective images. Confocal sections shown in (A) and (B) are 2.2 µm apart. (C-D'') Follicular epithelium of the Baz-GFP BAC line imaged at the focal plane of the adherens junctions (C-C'') and the nuclei (D-D'') stained with anti-Baz (C, D), GFP (C', D') and Hoechst (C'', D''). Junctional staining of anti-Baz colocalizes with Baz-GFP stained with anti GFP (C, C') whereas the nuclear envelope signal of anti-Baz does not show any overlap with Baz-GFP staining, which shows only background signal at the focal plane of the nuclei (D, D'). Hoechst staining in (C'', D'') is shown to demonstrate the focal plane of the respective images. Confocal sections shown in (C) and (D) are 2.4 µm apart. (E-F'') Follicular epithelium of the GFP-Baz protein-trap line imaged at the focal plane of the adherens junctions (E-E'') and the nuclei (F-F'') stained with anti-Baz (E, F), GFP (E', F') and Hoechst (E'', F''). Junctional staining of anti-Baz colocalizes with GFP-Baz stained with anti GFP (E, E') whereas the nuclear envelope signal of anti-Baz does not show any overlap with GFP-Baz staining, which shows only background signal at the focal plane of the nuclei (F, F'). Hoechst staining in (E'', F'') is shown to demonstrate the focal plane of the respective images. Confocal sections shown in (E) and (F) are 2 µm apart. Images are displayed in inverted gray scale. Scale bar in (A) = 20 µm, valid for all panels. Genotypes: (A, B) $w^{1118}$. (C, D) *w, P{CaryP, PB[BAC BazsfGFP2]attP18}* (on X). (E, F) *w, baz-GFP*$^{CC01941}$.

Biology Open • Supplementary information

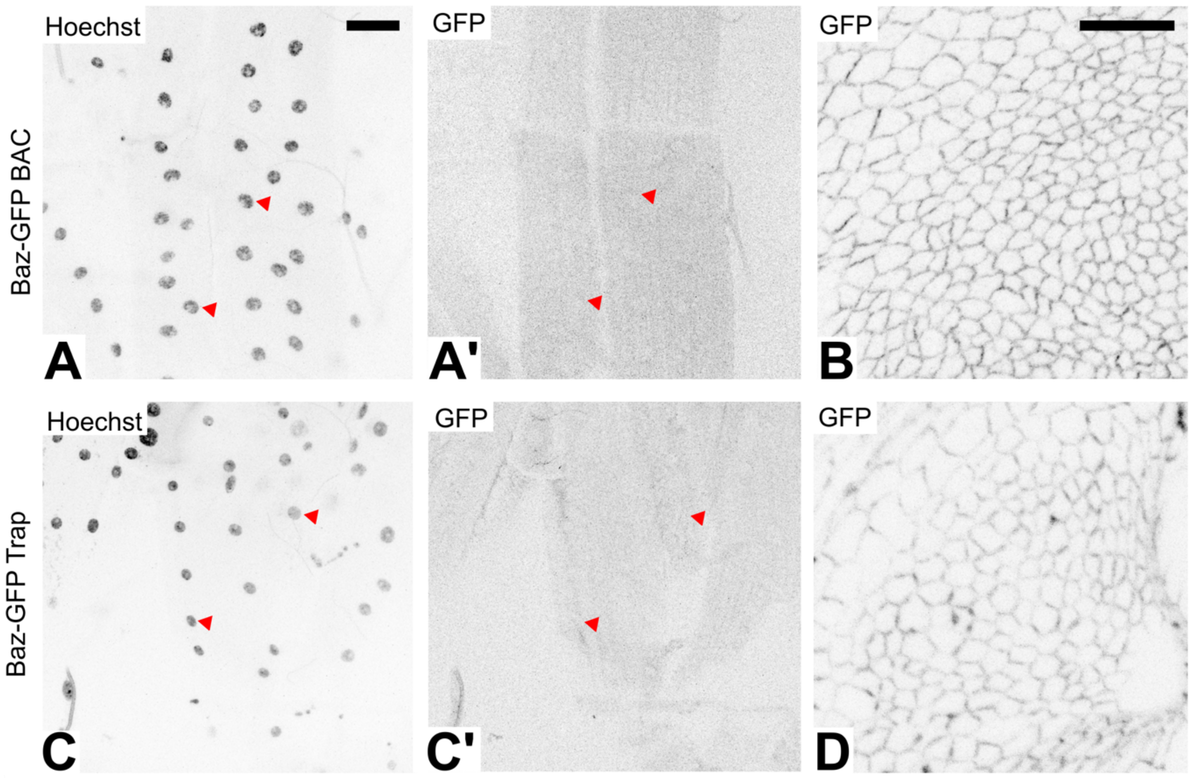


**Fig. S4. GFP fluorescence of Baz-GFP and GFP-Baz is undetectable in somatic body wall muscles.** (A-B) Baz-GFP expressed in the BAC line is undetectable in somatic body wall muscles (A') but shows junctional signal in a wing imaginal disc (B) from the same larva as shown in (A, A'). (C-D) GFP-Baz expressed in the trap line is undetectable in somatic body wall muscles (C') but shows junctional signal in a wing imaginal disc (D) from the same larva as shown in (C, C'). Larval tissue was fixed, stained with Hoechst (A, C) to select the proper focal plane containing nuclei for optical sectioning and imaged for endogenous GFP fluorescence without prior antibody staining (A', B, C', D). Red arrowheads in (A, A', C, C') mark nuclei. The settings of the confocal microscope were identical in (A', B, C', D). Images are displayed in inverted gray scale. Scale bar in (A) = 50 µm, valid for (A, A', C, C'), scale bar in (B) = 10 µm, valid for (B, D). All images are maximum intensity projections of confocal z-stacks. (A, A') 18 sections covering 20.5 µm, (B) 8 sections covering 2.7 µm, (C, C') 19 sections covering 21.5 µm, (D) 20 sections covering 7.3 µm. Genotypes: (A, B) *w, P{CaryP, PB[BAC BazsfGFP2]attP18}* (on X). (C, D) *w, baz-GFP$^{CC01941}$*.

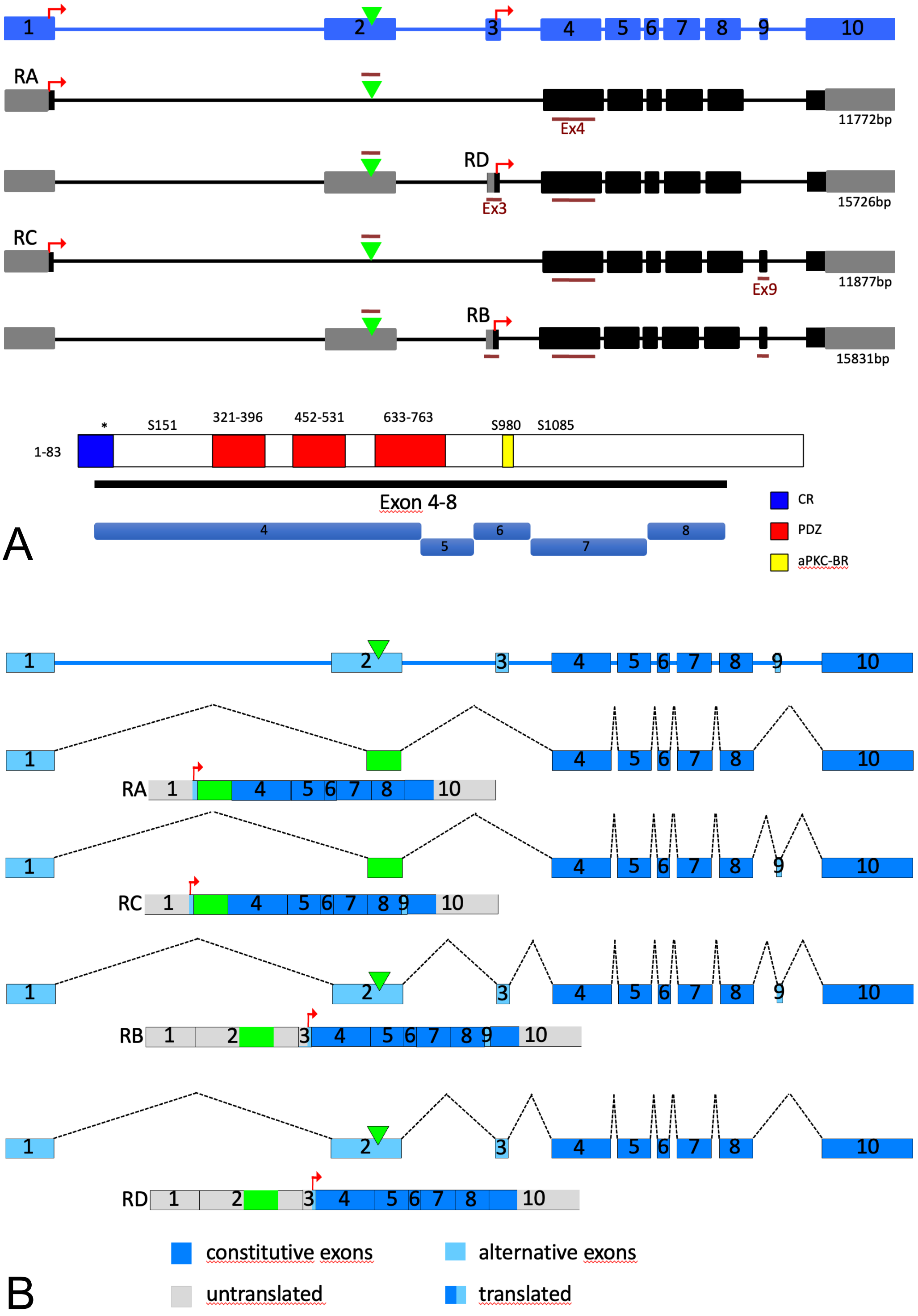


**Fig. S5. Genomic structure and transcripts of the GFP-Baz exon trap line (Buszczak et al., 2007).** (A) Genomic structure of the *baz* locus. Top: All exons of *baz* are indicated as blue boxes, introns are indicated as blue lines, the insertion of the artifical exon encoding GFP is

Biology Open • Supplementary information

marked by a green triangle. Middle: From the *baz* locus four different transcripts RA, RB, RC and RD can be generated. Exons contributing to each transcript are indicated as grey or black boxes. Untranslated exons or parts of exons are gray, translated exons or parts of exons are black. The translation start site is indicated by a red arrow in each transcript. Bottom: Domain structure of the Baz protein, isoform A with the exons encoding the respective protein domains shown at the very bottom. Note that with exception of the very N-terminus and the very C-terminus, all four isoforms of Baz have the identical domain structure encoded by the common constitutive exons 4-8. CR, conserved region 1; PDZ, Postsynaptic Density 95/Discs Large/Zonula Occludens 1 domain; aPKC-BR, atypical protein kinase C binding region. The asterisk above CR marks the position of the stop codon in *baz*$^{EH747}$. (B) Splicing patterns and structure of the corresponding mature mRNAs for the four different *baz* transcripts. Note that exons 2 and 3 are alternatively spliced exons that contribute only to transcripts RB and RD. The artificial exon encoding GFP within the untranslated exon 2 has strong splice acceptor and donor sites and therefore is constitutively spliced into all four isoforms. However, as translation of transcripts RB and RD is initiated in exon 3, GFP fusion proteins are only translated from transcripts RA and RC. Exon-intron-structure is not strictly drawn to scale.

Biology Open • Supplementary information

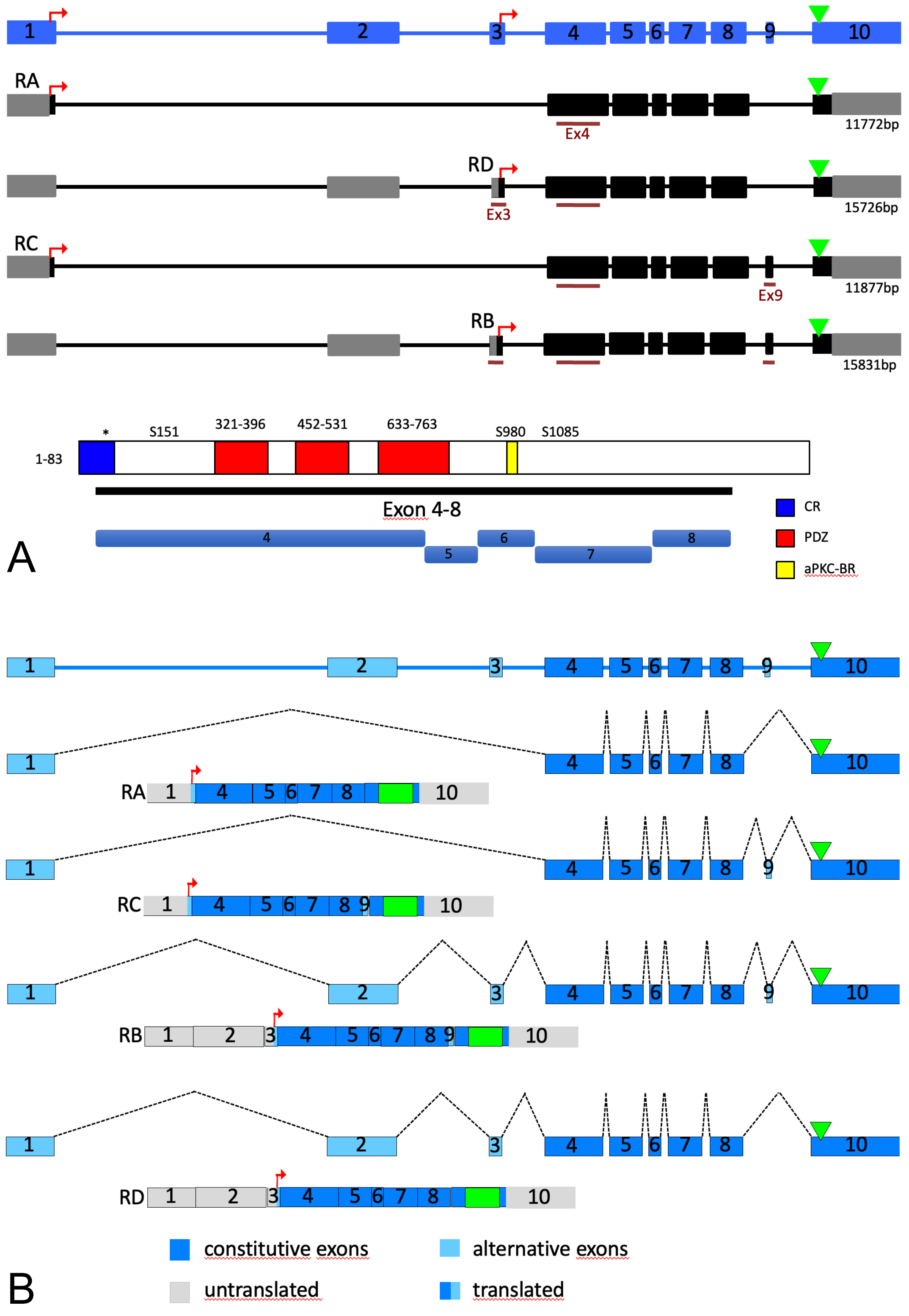


**Fig. S6. Genomic structure and transcripts of the Baz-GFP BAC (Besson et al., 2015).** (A) Genomic structure of the *baz* locus. Top: All exons of *baz* are indicated as blue boxes, introns are indicated as blue lines, the insertion position of the DNA sequence encoding GFP in exon

Biology Open • Supplementary information

10 is marked by a green triangle. Middle: From the *baz* locus four different transcripts RA, RB, RC and RD can be generated. Exons contributing to each transcript are indicated as grey or black boxes. Untranslated exons or parts of exons are gray, translated exons or parts of exons are black. The translation start site is indicated by a red arrow in each transcript. Bottom: Domain structure of the Baz protein, isoform A with the exons encoding the respective protein domains shown at the very bottom. Note that with exception of the very N-terminus and the very C-terminus, all four isoforms of Baz have the identical domain structure encoded by the common constitutive exons 4-8. CR, conserved region 1; PDZ, Postsynaptic Density 95/Discs Large/Zonula Occludens 1 domain; aPKC-BR, atypical protein kinase C binding region. The asterisk above CR marks the position of the stop codon in *baz*$^{EH747}$. (B) Splicing patterns and structure of the corresponding mature mRNAs for the four different *baz* transcripts. Note that exons 2 and 3 are alternatively spliced exons that contribute only to transcripts RB and RD. The DNA sequence encoding GFP within exon 10 is present and translated in all four *baz* isoforms. Exon-intron-structure is not strictly drawn to scale.

Biology Open • Supplementary information

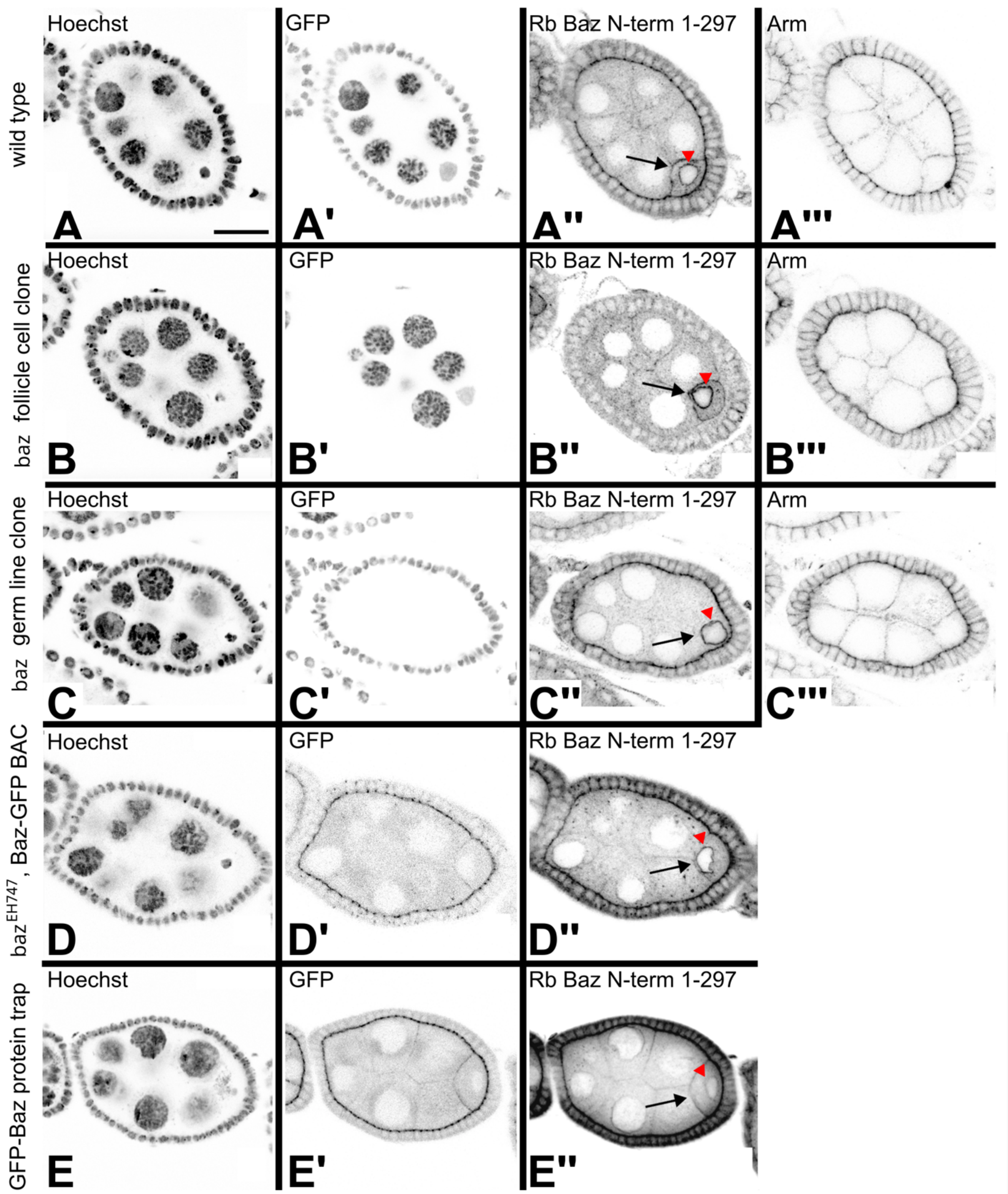


**Fig. S7. Nuclear envelope localization of Baz at the oocyte nucleus is a staining artifact.** (A-A''') Wild type egg chamber expressing nuclear GFP at stage 4/5 with the focal plane at the oocyte nucleus stained with Hoechst (A), GFP (A'), anti-Baz (A'') and Arm (A'''). Anti-Baz staining marks the apical adherens junctions of the follicular epithelium, the junctions between the germline cells and the nuclear membrane (A'', red arrowhead) of the oocyte (A'', arrow). Staining of Arm marks the apical junctions and the lateral membrane of the follicular epithelial cells and junctions between germline cells (A'''). (B-B''') Large follicle cell clone of *baz*$^{EH747}$. All nuclei are marked by Hoechst staining (B). Loss of nuclear GFP shows

Biology Open • Supplementary information

that the complete follicular epithelium is homozygous mutant for *baz*$^{EH747}$ (B'). Note that anti-Baz staining is lost at the apical junctions of follicular epithelial cells, but still visible in the germline, especially at junctions between oocyte and nurse cells (B'', arrow) and at the nuclear envelope of the oocyte (B'', red arowhead). Junctional staining for Arm is unaffected by loss of Baz in follicle cells (B'''). (C-C''') Germ line clone of *baz*$^{EH747}$. All nuclei are marked by Hoechst staining (C). Loss of GFP in germ line nuclei marks the *baz*$^{EH747}$ mutant germ line clone (C'). Anti-Baz staining marks the apical adherens junctions of the follicular epithelium, whereas the signal at the junctions between the germline cells is lost. However, the signal at the nuclear envelope of the oocyte persists (C'', red arrowhead). Junctional staining for Arm is unaffected by loss of Baz in germ line cells (C'''). (D-D'') Comparison of the signal using anti-Baz and anti-GFP antibodies in the Baz-GFP BAC line in the *baz*$^{EH747}$ mutant background. While anti-Baz immunostaining shows a signal at the nuclear envelope of the oocyte (D'', red arrowhead) in addition to junctional staining in the follicular epithelium and weakly between germ line cells (D'') the anti GFP antibody does not detect a signal at the nuclear envelope of the oocyte but only at the junctions in the follicular epithelium and weakly between the germ line cells (D'). (E-E'') Immunostaining using anti-Baz and anti-GFP in the GFP-Baz protein-trap line. Results are identical to those shown for the Baz-GFP BAC line. Anterior is to the left in all panels. Scale bar in (A) = 20 µm, valid for all panels. Genotypes: (A) *FRT19A/hsFlp*$^{122}$ *FRT19A H2AvD-GFP*. (B, C) *baz*$^{EH747}$ *FRT19A/hsFlp*$^{122}$ *FRT19A H2AvD-GFP*. (D) *w, P{CaryP, PB[BAC BazsfGFP2]attP18}, baz*$^{EH747}$*, FRT19A*. (E) *w, baz-GFP*$^{CC01941}$.

Biology Open • Supplementary information

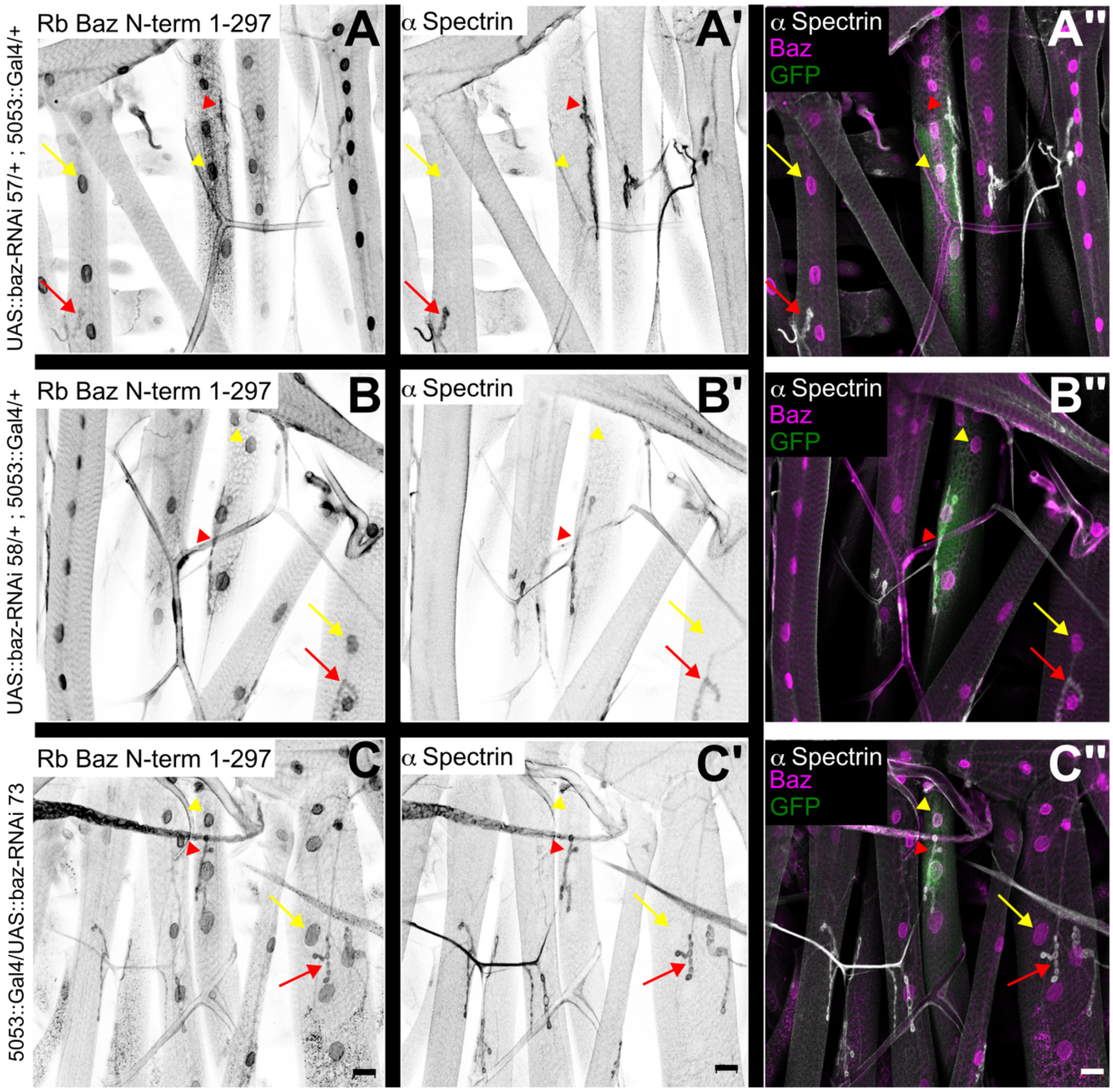


**Fig. S8. Baz RNAi at 29°C does not lead to reduced anti Baz staining at the nuclear envelope and the NMJ in somatic body wall muscles.** Three different RNAi constructs targeting *baz* were expressed in muscle M12 using the 5053::Gal4 driver line at 29°C when the UAS-Gal4 system is maximally active. (A-A'') Baz was downregulated using UAS::Baz-RNAi

57. Staining with rabbit anti Baz is shown in (A), α-Spectrin in (B) and the merged image plus GFP (green) in (A''). (B-B'') UAS::Baz-RNAi 58 was used to downregulate Baz in muscle M12. Stainings are as in (A-A''). (C-C'') UAS::Baz-RNAi 73 was used to downregulate Baz in muscle M12. Stainings are as in (A-A''). Yellow arrowheads mark nuclei in muscle M12, red arrowheads mark NMJs in muscle M12. Yellow arrows mark nuclei in muscles where Baz-RNAi is not expressed, red arrows mark NMJs in these muscles. Note that anti Baz staining intensity is not reduced in M12, nor is the staining intensity of α-Spectrin reduced compared to muscles not expressing Baz-RNAi. Scale bar in (C) = 20 µm, valid for all panels. Genotypes: (A) *UAS::baz-RNAi 57/+ ; 5053::Gal4 M12/+*. (B) *UAS::baz-RNAi 58/+ ; 5053::Gal4 M12/+*. (C) *5053::Gal4 M12/UAS::baz-RNAi 73* (on III.)

Biology Open • Supplementary information
